# Two weeks of exercise alters neuronal extracellular vesicle insulin signaling proteins and pro‐BDNF in older adults with prediabetes

**DOI:** 10.1111/acel.14369

**Published:** 2024-10-18

**Authors:** Steven K. Malin, Daniel J. Battillo, Michal S. Beeri, Maja Mustapic, Francheska Delgado‐Peraza, Dimitrios Kapogiannis

**Affiliations:** ^1^ Rutgers University New Brunswick New Jersey USA; ^2^ Division of Endocrinology, Metabolism & Nutrition Rutgers University New Brunswick New Jersey USA; ^3^ New Jersey Institute for Food, Nutrition and Health Rutgers University New Brunswick New Jersey USA; ^4^ Institute of Translational Medicine and Science Rutgers University New Brunswick New Jersey USA; ^5^ National Institute on Aging Baltimore Maryland USA

**Keywords:** aging, glucose tolerance, insulin sensitivity, obesity, physical activity, type 2 diabetes

## Abstract

Adults with prediabetes are at risk for Alzheimer's Disease and Related Dementia (ADRD). While exercise may lower ADRD risk, the exact mechanism is unclear. We tested the hypothesis that short‐term exercise would raise neuronal insulin signaling and pro‐BDNF in neuronal extracellular vesicles (nEVs) in prediabetes. Twenty‐one older adults (18F, 60.0 ± 8.6 yrs.; BMI: 33.5 ± 1.1 kg/m^2^) with prediabetes (ADA criteria; 75 g OGTT) were randomized to 12 supervised work‐matched continuous (*n* = 13, 70% HR_peak_) or interval (*n* = 8, 90% HR_peak_ and 50% HR_peak_ for 3 min each) sessions over 2‐wks for 60 min/d. Aerobic fitness (VO_2_peak) and body weight were assessed. After an overnight fast, whole‐body glucose tolerance (total area under the curve, tAUC) and insulin sensitivity (SIis) were determined from a 120 min 75 g OGTT. nEVs were acquired from 0 and 60 min time‐points of the OGTT, and levels of insulin signaling proteins (i.e., p‐IRS‐1, total−/p‐Akt, pERK1/2, pJNK1/2, and pp38) and pro‐BNDF were measured. OGTT stimulatory effects were calculated from protein differences (i.e., OGTT 60‐0 min). Adults were collapsed into a single group as exercise intensity did not affect nEV outcomes. Exercise raised VO_2_peak (+1.4 ± 2.0 mL/kg/min, *p* = 0.008) and insulin sensitivity (*p* = 0.01) as well as decreased weight (−0.4 ± 0.9 kg, *p* = 0.04) and whole‐body glucose tAUC_120min_ (*p* = 0.02). Training lowered 0‐min pro‐BDNF (704.1 ± 1019.0 vs. 414.5 ± 533.5, *p* = 0.04) and increased OGTT‐stimulated tAkt (−51.8 ± 147.2 vs. 95 ± 204.5 a.u., *p* = 0.01), which was paralleled by reduced pAkt/tAkt at 60 min of the OGTT (1.3 ± 0.2 vs. 1.2 ± 0.1 a.u., *p* = 0.04). Thus, 2 weeks of exercise altered neuronal insulin signaling responses to glucose ingestion and lowered pro‐BNDF among adults with prediabetes, thereby potentially lowering ADRD risk.

## Graphical abstract

Short‐term exercise training lowered fasting pro‐BDNF and increased glucose‐stimulated total Akt derived from neuronal extracellular vesicles (nEVs) in relation to peripheral insulin sensitivity among older adults with prediabetes.

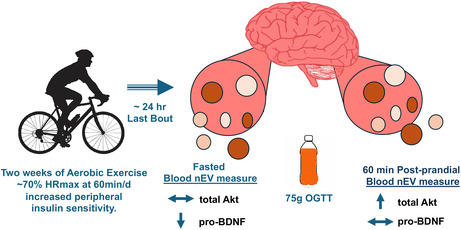

**Abbreviations**
ADA: American Diabetes Association
ADRD: Alzheimer's disease and related dementia
BDNF: brain derived neurotrophic factor
BMI: body mass index
HbA1c: hemoglobin A1c
HOMA‐IR: homeostatic model of insulin resistance
HR: heart rate
L1CAM: L1 cell adhesion molecule
nEV: neuronal extracellular vesicles
OGTT: oral glucose tolerance test
RER: respiratory exchange ratio
RPE: rating of perceived exertion
SiiS: simple index of insulin sensitivity
tAUC: total area under the curve
VO_2_ peak: peak oxygen consumption

## 1 INTRODUCTION

Prediabetes and type 2 diabetes have been linked to neuropsychological and neurologic diseases in humans, including major depression, cognitive decline, and Alzheimer's Disease and Related Dementia (ADRD) (Haroon et al., 2015). Insulin is typically discussed in terms of regulating peripheral tissue metabolism to maintain glucose homeostasis. However, insulin has neuronal functions since it can cross the blood–brain barrier and influence brain regions such as the prefrontal cortex and hypothalamus to impact cognition as well as memory (Kullmann et al., 2020). Aging and prediabetes have been linked to declines in insulin transport to brain regions, thereby raising questions on the impact peripheral insulin may have in ADRD pathogenesis (Arnold et al., 2018). A consequence of reduced insulin in the brain could result in decreased neuronal insulin signaling and/or brain insulin resistance that exacerbate cognition (Arnold et al., 2018). Subsequently, measuring brain insulin sensitivity has gained recent attention, and some have proposed use of intranasal insulin to assess functional changes through neuroimaging (Renner et al., 2012). Alternatively, neuronal extracellular vesicles (nEVs) are promising for providing insight to brain insulin sensitivity since nEVs can cross the blood–brain barrier for determination of insulin signaling proteins (e.g., IRS‐1, Akt, etc.) in the periphery (Dickens et al., 2017; Kapogiannis et al., 2015). Indeed, Kapogiannis et al. have shown that nEVs can be isolated from peripheral blood via immunocapture targeting the neuronal cell adhesion molecule L1CAM, but also that nEVs can be leveraged as a diagnostic tool for AD and other neurological disorders (Blommer et al., 2023; Pulliam et al., 2019). nEV markers of insulin resistance have also been reported in people with type 2 diabetes and Alzheimer's disease (Diehl et al., 2017). Thus, targeting insulin for brain health may have clinical implications since nearly 5 million Americans are living with Alzheimer's disease and almost 90 million have prediabetes (Matthews et al., 2019).

Exercise favorably influences cognitive function in individuals across the lifespan (Malin et al., 2022). This improved cognition has been related to maintenance/increase in gray matter volume (Broadhouse et al., 2020; Colcombe et al., 2006; Paillard, 2015), cerebral blood flow (Chung et al., 2018; Mazzoli et al., 2021; Paulus et al., 2021; Zhang et al., 2015), as well as elevated mitochondrial oxidative capacity and reductions in neuro‐inflammation (Ruegsegger et al., 2019). However, few data exist specifically determining the effects of exercise on brain insulin sensitivity, despite most work from rodents suggesting that exercise raises insulin signaling in the brain (Park et al., 2019; Ruegsegger et al., 2019). Interestingly, recent work (Kullmann et al., 2022) showed that exercise training for 8 weeks increased brain insulin sensitivity as measured by cerebral blood flow before and after intranasal insulin in young adults with excess weight. Consistent with this notion that exercise impacts insulin action in the brain is recent work demonstrating that aerobic exercise for 16 weeks raises nEV levels of brain‐derived neurotrophic factor (BDNF), pro‐BDNF, and humanin in patients with AD (Delgado‐Peraza et al., 2023). However, to date, no studies have examined whether exercise may impact nEV cargo as it relates to insulin signaling mediators and pro‐BDNF in older adults with prediabetes. Thus, we tested here the hypothesis that exercise would modulate nEV‐associated insulin signaling biomarkers and pro‐BDNF in relation to glucose tolerance and peripheral insulin sensitivity.

## 2 METHODS

### 2.1 Subjects

Twenty‐one adults (18F, 60.0 ± 8.6 yrs.; BMI: 33.5 ± 1.1 kg/m^2^) were part of our prior randomized‐controlled study comparing interval and continuous exercise on glucose tolerance (Gilbertson et al., 2018). This group of 21 adults studied herein were selected as they had plasma available for nEV assessment. There was no effect of intensity on respective nEV outcomes, nor outcomes related to glucose tolerance or insulin sensitivity as previously reported (Gilbertson et al., 2018). As such, we collapsed the two intervention groups to test the effect of exercise on outcomes. Participants were recruited via flyers and/or newspaper advertisements from the local community. All participants underwent a 75 g oral glucose tolerance test (OGTT) to determine prediabetes status (i.e., fasting glucose 100–125 mg/dL, 2 h glucose 140–199, and/or HbA1c 5.7%–6.4%) according to the American Diabetes Association (ADA) (Gilbertson et al., 2018). Participants were sedentary (exercise <60 min/wk) and did not smoke. They also underwent an examination that included a resting and exercise stress test with electrocardiogram. Blood and urine chemistry analyses were conducted to exclude people with known disease (e.g., type 2 diabetes, liver disease, cardiac dysfunction, etc.). Participants were excluded if taking medications considered to impact glycemia (e.g., biguanides, GLP‐1 agonists, etc.). All subjects provided written, signed, and verbal informed consent as approved by the Institutional Review Board.

### 2.2 Body mass and aerobic fitness

Weight was assessed on a digital scale, and height was recorded with a stadiometer to determine body mass index (BMI). Participants then completed a continuous incremental peak oxygen consumption (VO_2_peak) test using a cycle ergometer with indirect calorimetry (Carefusion, Vmax Encore, Yorba Linda, CA), and the heart rate (HR) peak was used to prescribe submaximal exercise.

### 2.3 Metabolic control

Participants were instructed to consume a diet containing about 250 g of carbohydrates during the 24 h. period prior to the pre‐intervention testing. This dietary pattern was recorded and replicated on the day before posttesting. Three‐day food logs, including two weekdays and one weekend day, were used to assess ad libitum food intake before and after training (ESHA Research, Version 11.1, Salem, OR). Participants were also instructed to refrain from alcohol, caffeine, medication, and strenuous physical activity for 24 h. prior to each study visit. Post‐intervention assessments were obtained approximately 24 h. after the last training session.

### 2.4 Exercise training

Participants were randomly assigned to 12, 60 min/d work‐matched bouts of continuous or interval cycle ergometry exercise over 13 days. A rest day was provided on day 7. Continuous exercise was performed at a constant intensity of 70% HRpeak, whereas interval exercise involved alternating 3‐min intervals at 90% HRpeak followed by 50% HRpeak. Subsequently, both interventions were designed to exercise at approximately 70% HRpeak, indicating all people underwent similar energy expenditure. HR (Polar Electro, Inc., Woodbury, NY) and rating of perceived exertion (RPE) were monitored throughout training to ensure intensity.

### 2.5 Glucose tolerance and insulin sensitivity

Following an approximate 10 h fast, participants reported to the Clinical Research Unit. Participants rested in a semi‐supine position for 15 min in which a ventilated hood was then used with indirect calorimetry for an additional 15 min to assess fuel use. The final 5 min were averaged to estimate breath samples (VO_2_ and VCO_2_) for respiratory exchange ratio calculations (i.e., VCO_2_/VO_2_). Then an intravenous line was placed in the antecubital vein for blood collection and determination of plasma glucose and insulin at 0, 30, 60, 90, and 120 min. nEVs were obtained at 0 and 60 min to determine glucose‐stimulatory effects on proteins related to insulin signaling and pro‐BDNF (*see Biochemical Analysis for details*). We selected 60 min to measure nEVs with thoughts this is generally near peak endogenous insulin levels. Total area under the curve (tAUC) during the OGTT was calculated using the trapezoidal rule. Peripheral insulin sensitivity was calculated as the simple index of insulin sensitivity (SIis) (Bastard et al., 2007), and hepatic insulin resistance was estimated by multiplying fasting glucose by fasting insulin divided by 405 as we have done before (Malin et al., 2018). Breath samples were also collected at 60 and 120 min and then averaged to calculate metabolic flexibility (i.e., average post‐prandial RER – fasting RER).

### 2.6 nEV isolation

nEVs were isolated following our immunoaffinity capture methodology targeting the L1 Cell Adhesion Molecule (L1CAM), a transmembrane neuronal protein. Our methodology has been characterized thoroughly in previous reports showing the purification of nEVs of expected size from free plasma components, as well as the enrichment of L1CAM+ EVs co‐carrying bonafide neuronal markers (Delgado‐Peraza et al., 2023; Vreones et al., 2023). Of note, volumes of all reagents were normalized to the initial plasma volume throughout the isolation protocol, thus ensuring that biomarker outcomes were not confounded by nEV recovery. Reagent volumes are expressed per 100 μL of plasma. First, plasma was defibrinated via addition of 1.2 μL of Thrombin (cat. no. TMEXO‐1; System Biosciences) and incubation at room temperature (RT) for 30 min, followed by dilution with 98.8 μL of Dulbecco's PBS 1‐X (DPBS) supplemented with 2X protease (cOmplete™ Protease Inhibitor Cocktail; cat no. 04693116001; Roche) and phosphatase inhibitors (Halt™ Phosphatase Inhibitor Cocktail; cat no. 78427; Thermo Fisher Scientific) and sedimentation at 6000 × g for 20 min at RT. The supernatant was transferred to a sterile microtube and total EVs were sedimented via incubation with 50.4 μL of Exoquick™ (cat no. EXOQ100A‐1; System Biosciences) for 60 min at RT, followed by centrifugation at 1500 ***g*** for 20 min at 4°C. The crude EV pellet was resuspended in 140 μL of ultra‐pure distilled water supplemented with 1X protease/phosphatase inhibitors overnight with gentle rotation mixing at 4°C. Crude EVs were incubated for 120 min at 4°C with 0.8 μg of biotinylated anti‐human L1CAM antibody (clone 5G3; cat. no. 13–1719‐82; Thermo Fisher Scientific) to derive nEVs. The EV antibody complexes were then incubated with 5 μL of washed Pierce™ Streptavidin Plus UltraLink™ Resin (cat. no. 53117; Thermo Fisher Scientific) for 60 min at 4°C with gentle rotation. After centrifugation at 600 ***g*** for 10 min at 4°C and removal of supernatant, nEVs were eluted with 40 μL of 0.1 M glycine (stock solution 1 M, pH 2.7; cat. np. 24,074–500; Polysciences, Inc.) and vortex mixed gently for 10 s. Beads were sedimented by centrifugation at 4500 ***g*** for 5 min at 4°C, and the supernatant containing immunoprecipitated nEVs was transferred to a clean tube, where pH was immediately neutralized with 6 μL of 1 M Tris‐Hydrochloride (pH 8; cat. no. CAS1185‐53‐1; Fisher Scientific). The produced volume was subjected to lysis via two freeze–thaw cycles in 10 μL of 3% bovine serum albumin (BSA) and 48 μL of mammalian protein extraction reagent (M‐PER)™ lysis buffer (cat. no. 78501; Thermo Scientific) supplemented with 2.2X protease/phosphatase inhibitor cocktail and stored at −80°C.

### 2.7 Biomarker determination in nEV lysates

We used an ELISA to quantify the concentration of proBDNF (cat. no. BEK‐2237; Biosensis, Thebarton, Australia) on a Synergy™ H1 microplate reader set to 450 nm and the Gen5™ microplate data collection software (BioTek Instruments). In addition, we used Meso Scale Discovery (MSD, Rockville, MD) to measure p‐IRS‐1^S312^ (cat. no. 150HLD), pERK1/2, pJNK, pp38 (MAP Kinase phosphoprotein panel; cat. no. K15101D), and pAKT (S473)/total AKT (cat. no. K15100D). These insulin signaling markers in the brain were selected since insulin acts mainly through MAPK as well as the IRS‐1‐PI3K‐Akt pathway to influence neuronal function (Kim & Feldman, 2012). Electrochemiluminescence assays were read using a MESO QuickPlex SQ120 imager and the Workbench Software 4.0 (MSD). nEV samples were run in duplicate with input based on experiments identifying the optimal sample dilution resulting in signals within the dynamic range of each assay. Standard curve equations both for ELISA and MSD assays were determined using the four‐parameter logistic (4‐PL) regression.

### 2.8 Clinical biochemical analysis

Plasma glucose was analyzed by a glucose oxidase assay (YSI Instruments 2700, Yellow Springs, OH). Remaining samples were centrifuged at 4°C for 10 min at 3000 RPM and stored at −80°C until later batched‐analyzed in duplicate to minimize variance within conditions. Insulin and nEV EDTA vacutainers contained aprotonin. Insulin was measured using an ELISA (cat. no. EXHI‐14 K, Millipore, Billerica, MA).

### 2.9 Statistical analysis

Data were analyzed using R (The R Foundation, Vienna, Austria 2013). Normality was assessed, and exercise training effects were compared using a paired, two‐tailed *t*‐test. Pearson's correlation was used to determine associations. Significance was set at *p* ≤ 0.05. Data are expressed as mean ± SD as well as delta scores (post minus pre) where appropriate.

## 3 RESULTS

### 3.1 Participant characteristics

Individuals on average had a HR of approximately 75 ± 0.8% and RPE (12.3 ± 0.3 a.u.) with an exercise energy expenditure (388.3 ± 14.8 kcal/session) throughout exercise training. This exercise dose reduced body weight (−0.43 ± 0.93 kg, *p* = 0.04) and raised VO_2_peak (1.4 ± 2.0 mL/kg/min; *p* = 0.008). There were no statistical differences in caloric or macronutrient intake following exercise training (Table 1).

**TABLE 1:** Body mass, aerobic fitness, and glucose metabolism before and after training.

	Pre	Post	Delta (∆)	*p*‐Value
*Demographics*				
Age (years)	60.1 ± 1.9	—	—	—
Sex (M/F)	(3/18)	—	—	—
Body weight (kg)	90.3 ± 3.4	89.8 ± 3.4	−0.4 ± 0.2	0.043
BMI (kg/m^2^)	33.4 ± 1.3	33.2 ± 1.3	−0.2 ± 0.1	0.049
Waist Circumference (cm)	105.5 ± 2.8	104.8 ± 2.7	−0.9 ± 0.6	0.118
VO_2_peak (mL/kg/min)	20.2 ± 1.1	21.5 ± 1.1	1.4 ± 0.5	0.008
*Cardiometabolic risk*				
SBP (mmHg)	127.7 ± 3.0	125.7 ± 2.9	−1.9 ± 1.8	0.283
DBP (mmHg)	71.8 ± 2.0	70.0 ± 2.2	−1.8 ± 1.2	0.163
MAP (mmHg)	90.4 ± 2.2	88.6 ± 2.2	−1.8 ± 1.0	0.097
Total Cholesterol (mg/dL)	203.0 ± 7.4	195.0 ± 7.1	−6.9 ± 4.0	0.107
LDL Cholesterol (mg/dL)	131.6 ± 6.4	127.3 ± 6.2	−3.1 ± 3.4	0.385
HDL Cholesterol (mg/dL)	52.6 ± 2.5	52.2 ± 2.1	−0.4 ± 0.9	0.641
Triglycerides (mg/dL)	114.9 ± 11.3	93.6 ± 7.9	−20.2 ± 6.3	0.004
*Glucose metabolism*				
Fasting Glucose (mg/dL)	103.3 ± 2.0	100.4 ± 1.9	−3.0 ± 1.6	0.078
2‐h Plasma Glucose (mg/dL)	151.6 ± 7.5	136.1 ± 8.3	−15.4 ± 6.7	0.031
Fasting Insulin (uU/mL)	98.6 ± 64.0	78.2 ± 48.4	−20.3 ± 42.2	0.038
2‐h Plasma Insulin (uU/mL)	98.6 ± 14.0	78.2 ± 10.3	−20.4 ± 9.2	0.039
Glucose tAUC (mg/dL‐120 min)	18,572 ± 872	17,123 ± 831	−1449 ± 582	0.021
Insulin tAUC (uU/mL‐120 min)	9958.7 ± 4905.9	8790.3 ± 4819.7	−1168.4 ± 2511.4	0.045
HOMA‐IR	3.1 ± 0.5	3.0 ± 0.4	−0.1 ± 0.2	0.672
SIis	0.193 ± 0.008	0.198 ± 0.011	0.004 ± 0.006	0.003
Fasting RER	0.81 ± 0.01	0.79 ± 0.01	−0.02 ± 0.01	0.024
Metabolic Flexibility	0.04 ± 0.01	0.05 ± 0.01	0.01 ± 0.01	0.199
*Dietary intake*				
Calories (kcal/d)	2210.2 ± 409.2	2118.9 ± 535.7	−91.3 ± 684.0	0.583
Carbohydrate (g)	267.8 ± 86.0	255.8 ± 54.9	−12.0 ± 81.0	0.600
Total Fiber (g)	26.6 ± 14.1	22.9 ± 6.3	−3.6 ± 13.6	0.301
Sugar (g)	108.2 ± 45.1	100.0 ± 25.0	−8.1 ± 40.2	0.484
Fat (g)	85.5 ± 26.7	88.5 ± 24.2	3.0 ± 39.2	0.743
Saturated Fat (g)	27.5 ± 12.5	28.9 ± 12.4	1.3 ± 17.3	0.743
Protein (g)	94.9 ± 41.5	80.4 ± 20.7	−14.5 ± 40.5	0.173

*Note*: Data are expressed as mean ± SD. The homeostatic model of insulin resistance (HOMR‐IR) was calculated as fasting PG × fasting PI, then divided by 405 to depict hepatic insulin resistance. SiiS = simple index of insulin sensitivity was calculated from plasma glucose and insulin to estimate peripheral or muscle insulin resistance. Dietary data is presented from 3‐day food logs before and after training.

Abbreviations: DBP, diastolic blood pressure; HDL, high density lipoprotein; LDL, low density lipoprotein; MAP, mean arterial blood pressure; SBP, systolic blood pressure; tAUC, total area under the curve.

### 3.2 Insulin sensitivity and glucose metabolism

Although hepatic insulin resistance (i.e., HOMA‐IR) was unaltered after the intervention (−0.10 ± 1.0 a.u., *p* = 0.67), peripheral insulin sensitivity (i.e., SIis) increased (0.004 ± 0.006 a.u., *p* = 0.003, Table 1). Training lowered fasting glucose (trend; −2.9 ± 7.8 mg/dL, *p* = 0.07) and postprandial glucose, as evident by decreased glucose tAUC_120min_ (−1448.8 ± 2665.6 mg/dL‐120 min, *p* = 0.02; Table 1). Fasting insulin levels were not different following exercise (*p* = 0.87), although training reduced insulin tAUC_120min_ (−1168.4 ± 2511.4 μU/mL‐120 min, *p* < 0.05, Table 1). Fasting RER was reduced (−0.02 ± 0.04, *p* = 0.02), reflecting greater fat oxidation. There was no change, however, in metabolic flexibility (0.01 ± 0.04, *p* = 0.19).

### 3.3 nEV insulin signaling and pro‐BDNF

**FIGURE 1:**
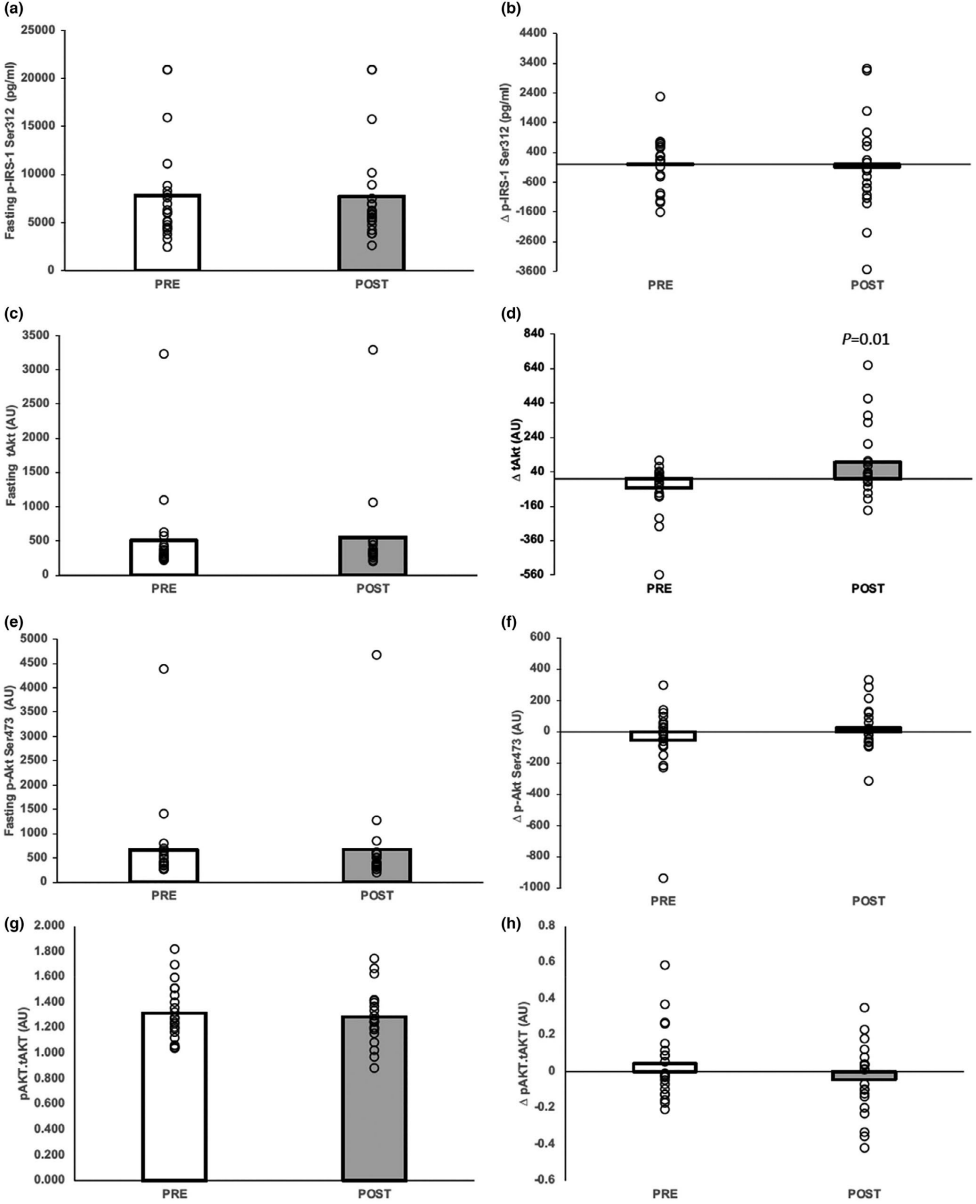
Impact of short‐term exercise training on neuronal extracellular vesicle‐derived IRS‐1‐PI3K‐Akt insulin signaling proteins. (a, c, e, and g) represent fasting levels. (b, d, f, and h) represent the change or delta (Δ) of insulin signaling proteins of the OGTT defined as 60 min minus 0 min. Bar graphs represent the mean with individual responses.

**FIGURE 2:**
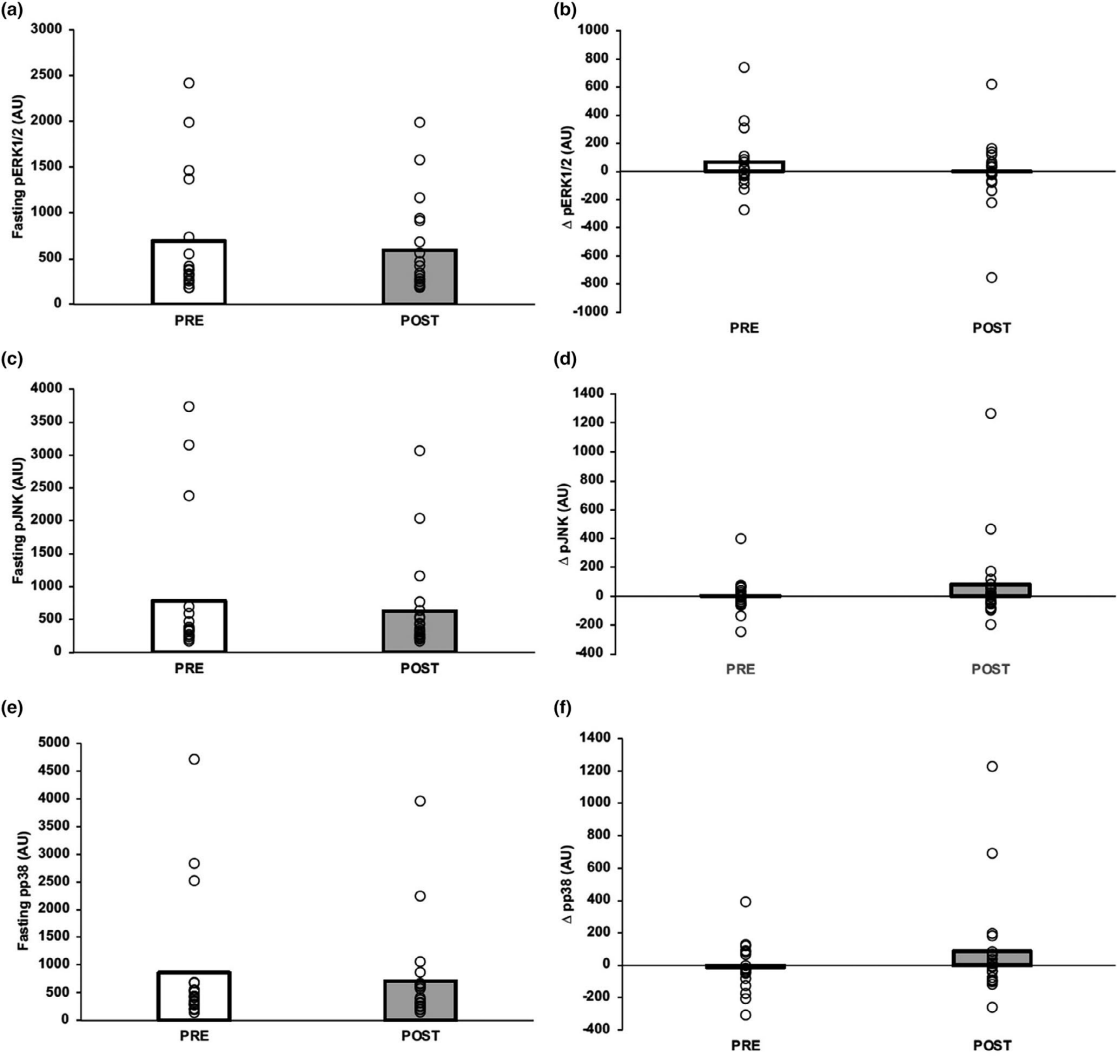
Impact of short‐term exercise training on neuronal extracellular vesicle‐derived Ras‐Mitogen Activated Protein Kinase (MAPK) insulin pathway. (a, c, and e) represent fasting levels. (b, d, and f ) represent the change or delta (Δ) of insulin signaling proteins of the OGTT defined as 60 min minus 0 min. Data were not normally distributed and log transformed for analysis. However, raw data are shown for ease of interpretation. Bar graphs represent mean with individual responses.

**FIGURE 3:**
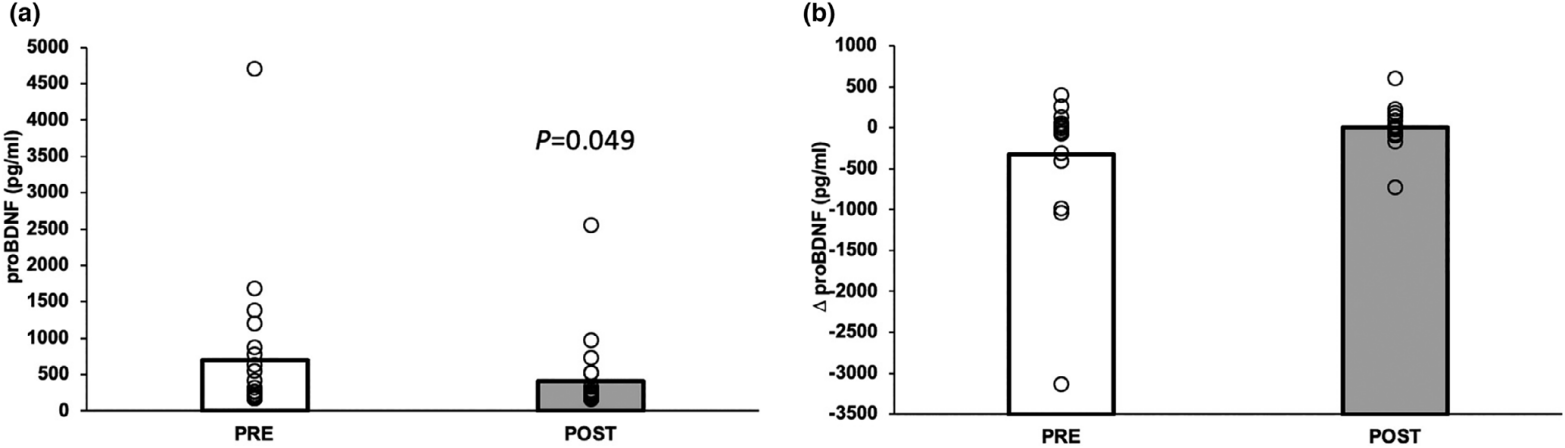
Impact of short‐term exercise training on neuronal extracellular vesicle‐derived pro‐BDNF. Measures were obtained at 0 min of the oral glucose tolerance test (OGTT) and the change in pro‐BDNF following glucose ingestion. The change or delta reflects 60 min minus 0 min of the OGTT. Bar graphs represent the mean with individual responses.

Exercise training had no effect on fasting p‐IRS‐1, pS473‐Akt, tAkt, pJNK, pERK1/2, or pp38 (Figures 1 and 2). Further, there was no effect on 60‐min levels or glucose‐stimulated responses in p‐IRS‐1‐Ser^312^, pS473‐Akt, pJNK, pERK1/2, or pp38 (data not shown). However, at 60 min, tAkt tended to rise following exercise (pre = 457.928 ± 534.910 vs. post = 545.596 ± 672.285, *p* = 0.065), while the ratio of pS473‐Akt to tAkt was lowered (pre = 1.358 ± 0.292 vs. post = 1.243 ± 0.187 a.u., *p* = 0.047). Further, the change in tAkt following glucose ingestion was statistically higher following exercise (*p* = 0.01, Figure 2). Fasting pro‐BDNF was lower after training (*p* = 0.049, Figure 3), whereas no effect was observed in changes in pro‐BDNF levels following glucose ingestion (*p* = 0.072, Figure 3).

### 3.4 Correlational analysis

**FIGURE 4:**
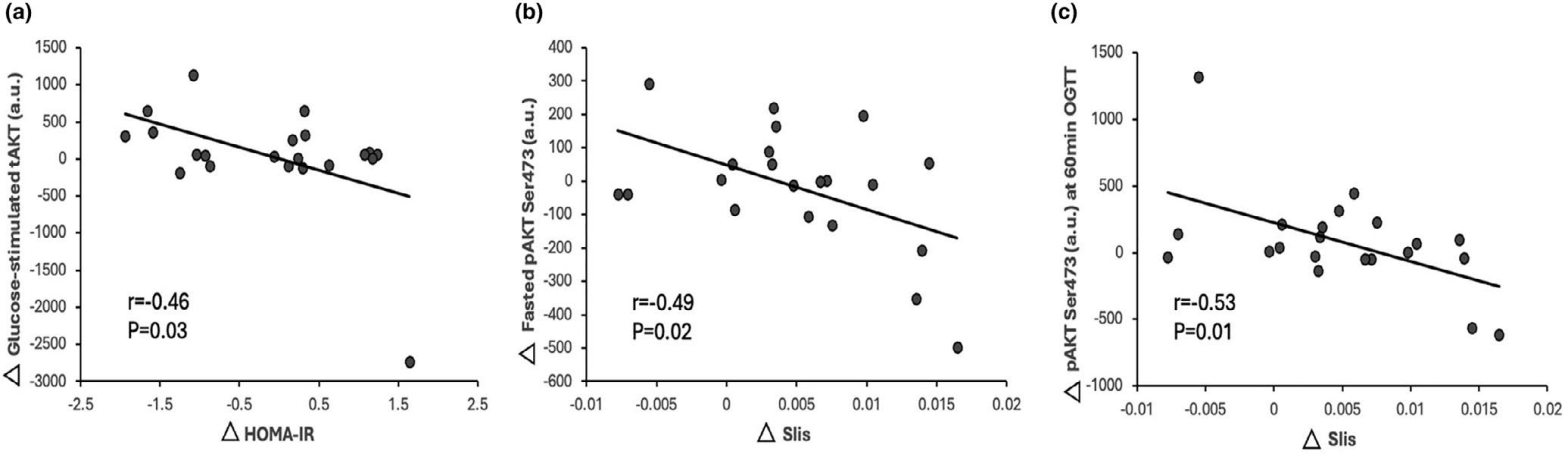
Correlation of neuronal extracellular vesicle‐derived insulin signaling proteins with peripheral insulin sensitivity.

Glucose‐stimulated pro‐BDNF responses were related to lower fasting RER (*r* = −0.44, *p* = 0.04). Increases in total Akt at 60 min of the OGTT after training correlated with reduced HOMA‐IR (*r* = −0.48, *p* = 0.02) and weight loss (*r* = −0.46, *p* = 0.03). Glucose‐stimulated tAkt after training related to weight loss (*r* = −0.47, *p* = 0.02), reductions in HOMA‐IR (*r* = −0.46, *p* = 0.03; Figure 4), and fasting plasma insulin (*r* = −0.45, *p* = 0.03). Fasting pAkt‐Ser^473^ reductions after training related to lower fasting glucose (*r* = 0.42, *p* = 0.05) and glucose AUC (*r* = 0.49, *p* = 0.02) as well as elevated peripheral insulin sensitivity (*r* = −0.49, *p* = 0.02; Figure 4). Reduced pAkt‐Ser^473^ at 60 min of the OGTT after training related to peripheral insulin sensitivity (*r* = −0.53, *p* = 0.01; Figure 4) and reduced pAkt‐Ser^473^ stimulation in response to glucose ingestion related to increases in VO_2_peak (*r* = −0.51, *p* = 0.01). Increased pAkt‐Ser^473^ to tAkt ratio at 60 min of the OGTT also related to lowered fasting RER (*r* = −0.47, *p* = 0.02). Weight loss was associated with reduced HOMA‐IR (*r* = 0.49, *p* = 0.02) but not peripheral insulin sensitivity (*r* = 0.00, *p* = 0.99). Higher VO_2_peak was related to lower fasting RER (*r* = −0.43, *p* = 0.05) only.

## 4 DISCUSSION

The main findings of the present study are that in older adults with prediabetes, short‐term exercise training raised nEVs derived tAkt during glucose stimulation. Interestingly, reductions in HOMA‐IR and body weight after exercise training were related to greater tAkt levels in response to glucose ingestion. In constrast, pAkt‐Ser^473^ was not altered in the fasting or postprandial state following exercise training, but the change in pAkt‐Ser^473^ during glucose ingestion after training related to gains in aerobic fitness as well as peripheral insulin sensitivity. Taken together, our findings highlight that short‐term training prior to clinically relevant weight loss is potentially able to improve brain insulin signaling among older adults with prediabetes.

We recently established that L1CAM+ nEVs are enriched for neuronal origin and offer a >20‐fold enrichment of neuronal material (Nogueras‐Ortiz et al., 2024). This firmly links the nEV cargo with their cells of origin, so that insulin signaling mediators in nEVs may be considered as a surrogate of the state of insulin signaling in brain neurons. From a pragmatic standpoint, these biomarkers can be used as predictive or diagnostic tools in various diseases with impaired insulin signaling, such as Parkinson's (Blommer et al., 2023) and Alzheimer's disease (Kapogiannis et al., 2015, 2019), but also as probes for studying neuronal cell physiology and its response to interventions in living humans (Kapogiannis et al., 2024), which is the main purpose of the present study. Thus, higher levels of phosphoproteins that indicate augmented insulin signaling in nEVs may be considered a surrogate of brain insulin sensitivity. Regarding the effects of Alzheimer's disease and type 2 diabetes or prediabetes on brain insulin resistance, preliminary evidence suggests that Alzheimer's disease can affect more drastic changes in nEV biomarkers than diabetes alone (Kapogiannis et al., 2015), but more research is required to delineate their complex interplay. This study for instance revealed associations between exercise‐induced changes in neuronal insulin signaling, as reflected in nEV levels of tAKT, and estimates of peripheral insulin sensitivity (i.e., HOMA‐IR and SiiS). While this association does not imply causation (i.e., we do not claim that nEV changes cause change in peripheral insulin sensitivity), we have also observed correlations between nEV‐associated t‐Akt and other biomarkers of insulin signaling with HOMA2‐IR at baseline and in response to two diets (Kapogiannis et al., 2024). Moreover, this relation is consistent with prior exercise work (Honkala et al., 2018) showing that exercise‐induced increases in peripheral insulin sensitivity are associated with brain insulin‐stimulated glucose uptake decreases.

On a molecular level, insulin signals in the brain occur mainly through two pathways: Ras‐Mitogen Activated Protein Kinase (MAPK) as well as the IRS‐1‐PI3K‐Akt pathway (Kim & Feldman, 2012). However, no prior work had examined how exercise impacted these pathways in humans. In an effort to close this knowledge gap, our results show that none of the MAPK‐related proteins p‐ERK1/2, p‐JNK, or pp38 were altered in the fasted or glucose‐stimulated state following short‐term training. The clinical ramifications of this are difficult to discern given the short‐term nature of the study, but typically MAPK pathways are linked to cell growth and proliferation as well as vasoconstriction (Kim & Feldman, 2012). Conversely, in the present study, exercise training seems to have impacted glucose‐stimulated tAkt in nEVs. This is potentially thought‐provoking since there was no effect of exercise on fasting tAkt. Prior work has shown that using an euglycemic hyperinsulinemic clamp is an effective means for raising central nervous system insulin concentrations (Fishel et al., 2005). Although we did not infuse insulin via the clamp approach here, it is reasonable from our data to suggest that neurons responded to the rise in insulin following glucose ingestion after short‐term training to elicit increases in tAkt compared with pre‐training. In fact, our findings are consistent with a recent exercise training study showing elevated intranasal insulin‐stimulated cerebral blood flow in young adults (Kullmann et al., 2022). Thus, more work is needed to understand how nEV insulin signaling biomarkers may relate to aging and diabetes‐related changes in the blood brain barrier and the transport of insulin from the periphery to the brain.

Why exercise training herein only upregulated tAkt but not p‐IRS‐1‐Ser^312^ or pAkt‐Ser^473^ is beyond the scope of the present work. Nonetheless, consideration of brain insulin signaling is of interest since insulin resistant adults have been reported to have higher brain glucose uptake in response to insulin than healthy controls (Hirvonen et al., 2011). It has been suggested that young and cognitively healthy individuals show maximum glucose uptake during physiologic fasting states, and their brain glucose uptake does not increase with insulin stimulation. However, insulin resistant individuals likely require more insulin (i.e., insulin stimulation) to match the normal brain glucose uptake seen in healthy adults. Among individuals with Alzheimer's disease, higher HOMA‐IR is associated with lower glucose uptake across brain areas (Willette et al., 2015). These observations are clinically relevant because insulin's action through pleiotropic (including trophic) effects, on neuronal cells is critical for memory, whereas reduced neuronal insulin signaling is a risk factor and pathogenically implicated in ADRD (Arnold et al., 2018). Intriguingly, Honkala et al. (2018) had demonstrated that 2‐weeks of sprint interval training, but not moderate‐intensity continuous exercise, decreased insulin‐stimulated glucose uptake in cortical gray matter in all brain regions except the occipital lobe in insulin‐resistant, middle‐aged adults. Moreover, this reduction in insulin‐stimulated brain glucose uptake related to higher peripheral insulin sensitivity as measured by the clamp. This prior finding is consistent with our observation of reduced pAkt‐Ser^473^ in the fasting and 60‐min timepoint of the OGTT as well as rise in peripheral insulin sensitivity. Further, we noted that reductions in HOMA‐IR after exercise related to rises in tAkt during the OGTT. Taken together, these findings suggest a regulation of peripheral tissues with neuronal insulin signaling for whole‐body insulin sensitivity homeostatsis. Additional work here is warranted to understand if and how nEV cargos relate to peripheral glucose metabolism (including that of skeletal muscle). Another consideration here is that the brain is typically prefers glucose as a fuel source in healthy individuals. However, during periods of energy deficit, such as weight loss, there are more free fatty acids liberated from adipose tissue, and the liver upregulates the production of ketone bodies. Both free fatty acids and/or ketones may serve as a fuel sources for tissues such as the brain (Honkala et al., 2018) during periods of weight loss, which in our study is most likely driven by increased energy expenditure since we observed no change in habitual diet intake. Interestingly, we observed in this study that reductions in RER were associated with lower pAkt‐Ser^473^/tAkt ratio during the OGTT after training. Given HOMA‐IR correlated with weight loss in the current study, these results are consistent with views that the brain mitochondrial oxidative capacity may have increased in relation to increased aspects of neuronal insulin signaling.

It is important to acknowledge that tAkt reflects a family of proteins: Akt 1/2/3. Thus, the exact meaning behind rises in tAkt is unclear, although pAkt‐Ser^473^ is considered key for full Akt activation for cogntive benefit (Gabbouj et al., 2019). As such, it is worth noting that the observed increase in tAkt with exercise training while pAkt‐Ser^473^ remained unaltered could imply relatively diminished feed‐forward insulin signaling. However, pAkt‐Thr^308^ could independently influence aspects of brain insulin signaling (Vanhaesebroeck & Alessi, 2000). For example, the rise in tAkt is of potential interest because it could inhibit GSK‐3β, a major tau phosphorylation regulator implicated in Alzheimer's disease progression (Takashima, 2006). Another possible factor explaining the rise in tAkt and lack of change in pAkt‐Ser^473^ relates to proBDNF. Indeed, proBDNF activates Akt during periods of hyperglycemia (Zhong et al., 2019). In turn, the lack of pAkt‐Ser^473^ activity after training may in part be explained by a tendency of lower fasting proBDNF and/or low glucose levels following exercise. The reductions in proBDNF observed in our study are of interest given that BDNF is a neurotrophic factor that is considered key for synaptic plasticity, growth and differentiation of neurons, neuroprotection, as well as learning and memory. Although it is suggested that exercise can raise BDNF levels (Cho & Roh, 2016), mixed results exist including no change (Enette et al., 2020; Goekint et al., 2010) or decrease in BDNF following exercise (Damirchi et al., 2014). The reasons for such discrepancy are difficult to determine but likely relate to the exercise regimens prescribed, the study populations, the heterogeneity of tissues releasing/sequestering BDNF, and/or the assessment of total versus active BDNF. In either case, elevations in BDNF have been suggested in prior exercise studies to benefit cognition through in part mitochondrial adaptations (Swain et al., 2023). Consistent with this, we noted that reductions in RER correlated with training induced rises in aerobic fitness and glucose‐stimulated pro‐BDNF in the present work. Obesity is also recognized as an inflammatory inducing state, and it has been reported that BDNF may be elevated in some older adults with obesity/metabolic syndrome as a compensatory mechanism to protect the brain from an inflammatory insult (Babaei et al., 2013). In our study, weight loss was less than 1 kg, and we observed no correlation of changes in pro‐BNDF with weight loss after training. Thus, alternative mechanisms may alter proBDNF levels. While we did not design this study to assess the exact mechanism(s) by which BDNF and proBDNF affect insulin signaling, these data provide rationale for additional work to understand how growth factors may relate to brain insulin action after exercise.

Afternoon exercise training has gained recent attention since it has been linked to better glucose metabolism and insulin sensitivity among people with obesity (Remchak et al., 2021). We allowed people to self‐select their exercise times while supervised in this study. Given this prior literature on the timing of exercise and health outcomes, we decided to conduct an exploratory analysis and categorized people by morning (*n* = 10, 6–10 AM), midday (*n* = 7, 11‐3 PM), or afternoon (*n* = 4, 3–7 PM) exercise training (for at least 70% of the time) on nEV insulin signaling. We report that morning exercise favored elevated p‐IRS‐1‐Ser^312^ in response to the OGTT (Figure S1), while morning and mid‐day exercise showed elevated tAkt at 60 min of the OGTT after training. Interestingly, those who exercised in the afternoon showed a lower ratio of pAkt‐Ser^473^ to tAkt after training. Despite the relatively small sample size, this observation implies that exercise time of the day might help explain variance in brain adapations among outcomes in our study that may prevent us from detecting changes across the entire study population. Further work is warranted to confirm and examine whether exercise time of day impacts brain insulin signaling and cognitive benefit.

This study has limitations that could impact our interpretations. We studied a relatively small sample size, and we are not powered to discern the potential impact of sex on respective outcomes. Therefore, we cannot generalize findings from this intervention across different populations. For instance, it is unclear how people in our study matched on age, sex, BMI, and fitness to adults without prediabetes would compare in terms of nEV insulin signaling before and after exercise training. Furthermore, we did not have a true control group (i.e., a non‐exercising group followed over the same period of time). However, we interpret the lack of change in fasting nEV biomarkers to serve as a proxy control. We used the OGTT to assess glucose‐stimulated insulin effects on nEV biomarkers, and it is possible that this approach may over−/under‐estimate effects on nEVs compared with intravenous insulin infusion methods. While use of the OGTT provides ecologically valid findings with physiologic relevance, we recognize that further work using insulin clamp/spray approaches would provide greater confidence in attributing effects to insulin per se. We also acknowledge that nEV insulin signaling proteins do not directly measure brain insulin sensitivity, although evidence from us and others support that these nEV bioamarkers may act as surrogates of insulin signaling in the parent cells (neurons). Treated as a “liquid biopsy,” nEVs offer insights into physiologic and pathologic (e.g., ADRD mechanisms) mechanisms operating in the cell and organ of origin. Indeed, we employed optimal preparations and sensitive single‐EV detection tools to identify neuronal‐specific epitopes on intact EVs. This study was of short‐term training, and additional work is warranted to understand how other exercise approaches (e.g., acute bouts/long‐term training, different modes, etc.) may influence nEV biomarkers. We did not measure cognition or brain function as part of this study, which limits the inferences on the functional impact of nEV biomarker changes we can make. Future cognitive performance studies with nEVs are needed in normal and diseased populations as well as additional studies assessing the effects of exercise on nEV biomarkers in normal and diseased populations. Nonetheless, the relation of nEV insulin signaling with peripheral measures of insulin sensitivity, weight loss, and fitness help infer clinical relevance. Moreover, a notable strength of this study is that it provides novel insight to how exercise may affect neuronal insulin signaling in relation to peripheral insulin sensitivity among a clinically relevant group of older adults with prediabetes who at risk for ADRD. Thus, this prospective ad hoc study underscores the relevance of nEVs and lays the foundation for additional studies of exercise on nEVs to enhance precision health for ADRD prevention and/or treatment.

In conclusion, short‐term exercise training increased nEV tAkt responses to a glucose load in older adults with prediabetes. However, exercise training had no effects on nEV‐associated p‐IRS‐1‐Ser^473^, pJNK, p‐ERK‐1/2, or pp38. Together, with improvements in systemic glucose tolerance and peripheral insulin sensitivity, these data suggest that exercise may influence brain insulin sensitivity through changes in insulin signaling. Further work is warranted to confirm if exercise raises brain insulin sensitivity as well as understand how improvements in nEV‐associated insulin signaling biomarkers relate to cognition and brain function to improve interventions designed to prevent, treat, and/or delay the onset of dementia in people at risk for type 2 diabetes.

## AUTHOR CONTRIBUTIONS

SKM conceptualized the overall study question and design. DK contributed to conceptualization of nEV protocol and analysis. DJB, MSB, MM, and FDP were responsible for data management, analysis and/or interpretations. SKM was primarily responsible for statistical analysis and wrote the manuscript. All authors edited the work and approved the final draft.

## FUNDING INFORMATION

Work supported by National Institutes of Health RO1‐HL130296 (SKM).

## CONFLICT OF INTEREST STATEMENT

The authors declare no conflicts of interest.

## Supporting information


Figure S1.

## Data Availability

Data will be made available upon reasonable request after correspondence with SKM.
