# Supplementary material for: Two weeks of exercise alters neuronal extracellular vesicle insulin signaling proteins and pro‐BDNF in older adults with prediabetes
Source: Aging Cell. 2024 Oct 18;24(1):e14369. doi: 10.1111/acel.14369 (PMC11709104; doi:10.1111/acel.14369)
Supplement: Supplementary file 1 — Figure S1. [file ACEL-24-e14369-s001.docx]

***Supplemental Figure 1.***

***
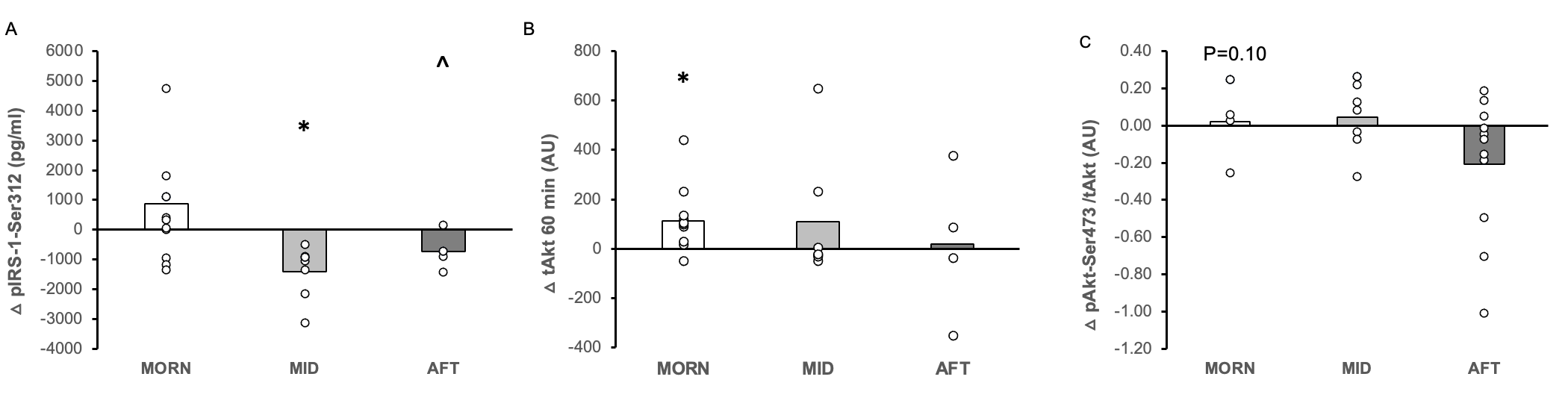
***

Exploratory analysis exercise time of day on neuronal extracellular vesicle derived canonical insulin signaling proteins. *Compared with Pre-test, *p* < 0.05. ^Compared with MORN, *p* < 0.05. Bar graphs represent mean with individual responses.
